# Assuring Primary Healthcare Services to Vulnerable Children in a Disadvantaged Suburb of Rome Metropolitan City During the Pandemic: Responses to the Crisis

**DOI:** 10.3390/children12040443

**Published:** 2025-03-30

**Authors:** Aurelia Rughetti, Anna Rita Buonomini, Leonardo Russo, Francesca Mazzoli, Suleika Urbano, Fotinì Iordanoglou, Cataldo Palagiano, Manuel Barletta, Samuele Casartelli, Aldo Morrone, Lucia Ercoli

**Affiliations:** 1 Solidarity Medicine Institute Onlus, Via Aspertini, 520, 00133 Rome, Italy; annarita.buonomini@gmail.com (A.R.B.); leonardodottrusso@gmail.com (L.R.); francesca.mazzoli@gmail.com (F.M.); suleikau@libero.it (S.U.); fotini.iordanoglou@gmail.com (F.I.); cataldo.palagiano@gmail.com (C.P.); manu.barlet@gmail.com (M.B.); lucia.ercoli@uniroma2.it (L.E.); 2 Department of Experimental Medicine, Sapienza University of Rome, Viale Regina Elena, 324, 00161 Rome, Italy; 3 Department of Sociology, Catholic University of Sacred Hearth, Largo A. Gemelli 1, 20123 Milan, Italy; samuele.casartelli@unicatt.it; 4 Istituto Internazionale di Scienze Mediche Antropologiche e Sociali, Via Tuscolana 120, 00173 Rome, Italy; aldomorrone54@gmail.com; 5 Department of Biomedicine and Prevention, Tor Vergata University, Via Cracovia, 50, 00133 Rome, Italy

**Keywords:** health equity, primary health care, COVID-19, BMI, poverty, mental health, urban suburb, children, adolescent

## Abstract

**Background/Objective:** This retrospective observational study describes the social, health, and psychological conditions of children living in a disadvantaged and degraded suburb of Rome Metropolitan City during the COVID-19 pandemic as registered by the primary healthcare service of the Solidarity Medicine Institute, with the aim of fighting social exclusion and health disparities during lockdown and offering free health care to vulnerable families. **Methods:** The access to pediatric interventions was assessed from April 2020 to December 2022. For each child, biometric parameters were recorded, and the physical and psychological states of health were assessed. Furthermore, data regarding family socio-economic variables were collected. **Results:** From April 2020 to December 2022, 638 children, aged 0 to 18 years, had access to the healthcare system, which was provided by the Solidarity Medicine Institute, with a total of 2300 pediatric visits. Moreover, food supplements, drugs, and hygiene kits that were necessary for the containment of the COVID-19 infection were freely distributed at the center. The highest proportion of children included in this study were from African and Eastern European families (46% and 35.8%, respectively), and 41% of these children did not have a pediatrician from the public health service. Children aged 0 to 5 years comprised 50.81% of the entire population of this study. Nutritional status assessment indicated that among the 117 infants aged 0–12 months, 5.7% were below the 3rd weight percentile, while 28.9% exceeded the 85th weight percentile. BMI assessment for children aged 2 years and older (i.e., 521 children) indicated that 21.7% of these children were overweight, and 9.5% were obese. Sixty-nine cases of psychiatric disorders were also detected among these children, with a high frequency of cases of Specific Language Disorder (31.8%), Attention Deficit Hyperactivity Disorder (21.7%), and Specific Learning Disorder (14.5%). Psychiatric and rehabilitative interventions were also offered. **Conclusions:** The Solidarity Medicine Institute responded to the request of the municipality of Rome to remain open and offer social and health assistance to the most vulnerable people during the pandemic. The Solidarity Medicine Institute has efficaciously served a fragile pediatric population, intercepting social, health, and psychological needs and overcoming social exclusion, health disparity, and the fragmentation of welfare services exacerbated by the COVID-19 pandemic.

## 1. Introduction

Children’s health care is a key issue of public health equity. The Convention on the Rights of the Child (General Assembly, UNO, 1989) [1] sanctioned children’s rights so that they possess the best possible state of health and benefit from medical and rehabilitation services. The monitoring of children’s growth and development, delivery of routine check-ups, and provision of counseling support to their parents/caregivers are essential interventions to guarantee children’s rights. Disappointingly, real-word experiences indicate that economic, social, cultural, and historical factors impede full access to public healthcare services, even in developed Western countries.

Furthermore, the COVID-19 pandemic has completely overturned the healthcare system. Indeed, the implementation of widespread lockdowns and restrictions impacted primary care service access and provision, with the overall impediment to pediatric health care [2]. These detrimental effects were magnified for the vulnerable populations of both low-income and developed countries [3,4]. During the COVID-19 pandemic, the number of children living in disadvantageous socio-economic conditions was increasing in our population, with important implications for physical and psychological development, in addition to the risk of greater future inequalities [5,6,7]. During home confinement, children are likely to live an unhealthy lifestyle characterized by sleep disturbances, less physical activity, and longer media exposure [8]. Several studies have raised concerns regarding the negative effects of prolonged school closures and home confinement on child well-being, during which children are more vulnerable to mental health problems [9]. Socio-economic inequality in child mental health was high during the pandemic, as an unhealthy lifestyle and an unfavorable family environment are contributory factors for child mental health problems [10]. It is known that children living in deprived suburban areas can experience stress, violence, and an increased risk of developing behavioral problems.

During the pandemic, these conditions were exacerbated, and access to health services was very low, especially for the most disadvantaged sections of the population. For most of them, there was no health care due to the temporary closure of the offices of general practitioners and pediatricians. Furthermore, the low educational levels of this segment of the population had dramatically impacted the possibility of them being adequately informed about the minimal health-related safety indications that were given, i.e., what should be done and what should not be done. In the public health emergency context, street units, as well as safety-net healthcare centers, have played crucial roles in containing the pandemic and guaranteeing health care [11,12] in line with the UN Agenda 2030 and the Sustainable Development Goal numbers 3 and 10 that ensure that “*no one is left behind*” [13]. The Solidarity Medicine Institute is a non-profit organization, active since 2003, that offers free, low-threshold medical services as well as psychological, primary socio-educational care in one of the most socially disadvantaged areas in the suburbs of Rome. It intercepts the needs of nuclear families, in particular, children and mothers, providing health care (therapeutic and prophylactic) and life-related support [14,15,16]. Furthermore, it offers free educational interventions for children and adolescents. During the pandemic, the Solidarity Medicine Institute was constantly active, providing services to a vulnerable population that had no access to public healthcare services, guaranteeing healthcare services, distributing personal protective equipment for daily life, and promoting disease prevention [17].

**Figure 1:**
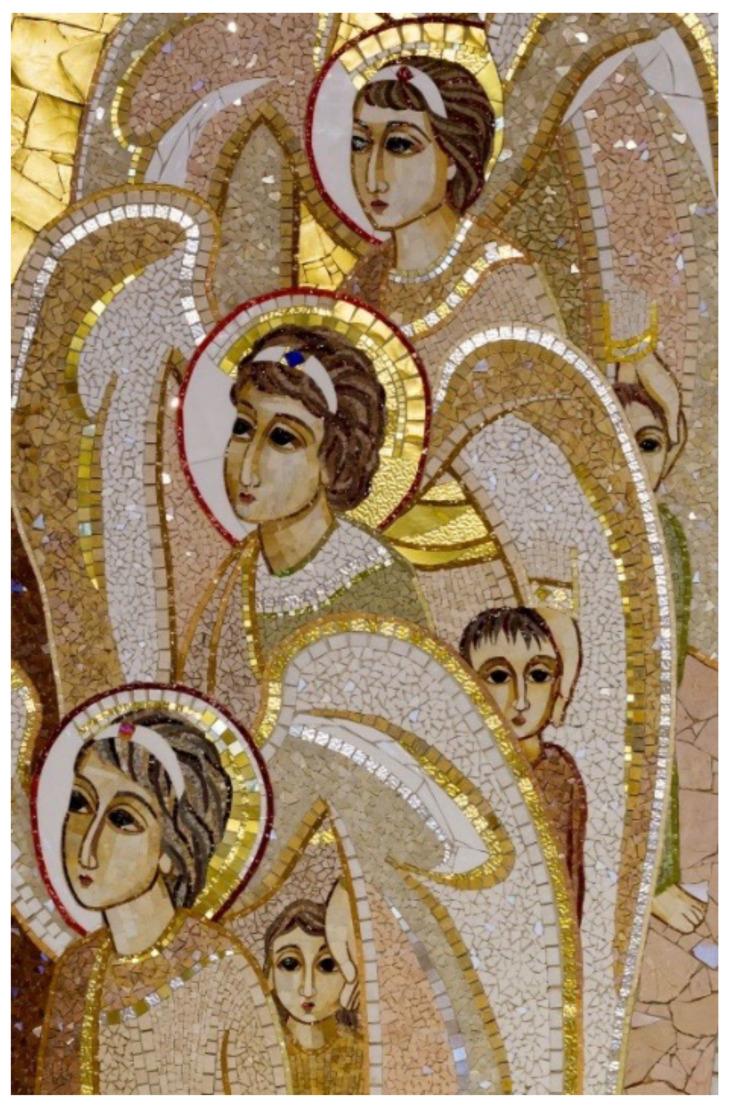
The *Guardian Angels* mosaic from the Church of Guardian Angels, Budapest, Hungary. This mosaic symbolically represents the function of primary healthcare services in disadvantaged communities, in particular, for vulnerable children.

This retrospective observational study describes the social determinants, health/illness state, and psycho-physical health of pediatric patients living in conditions of extreme fragility in the degraded suburbs of Rome during the pandemic period (April 2020–December 2022). The results underline the key role played by outreach clinics in providing primary healthcare and social services to fragile populations, especially during a global crisis (Figure 1).

## 2. Materials and Methods

### 2.1. Description of the Solidarity Medicine Institute

The Solidarity Medicine Institute (https://medicinasolidale.org (accessed on 29 March 2025)) is a primary care center for disadvantaged people located at the eastern/southeastern boundaries of Rome metropolitan city, just outside the major highway around Rome. Medical, psychological, and primary social care supports are offered to disadvantaged families, mainly children and women. The staff are made up of medical professionals (general physicians, pediatricians, child neuropsychiatrists, otolaryngologists, gynecologists, psychologists, nurses, etc.) and cultural mediators and educators. The healthcare center of the Solidarity Medicine Institute is open from 8.30 am to 4 pm from Monday to Friday, and from 8.30 am to 12.30 am on Saturday. The healthcare staff offer free services to the population, like primary health visits for diagnostic assessment, psychological support, primary social care support, and educational services.

Access to the Solidarity Medicine Institute’s services is free of charge and is provided independent of ethnicity and social status. Their services are mainly advertised by word of mouth among the people coming from the neighborhood as well as from other distant areas of Rome metropolitan city.

### 2.2. Collection of the Data and the Design of Retrospective Observational Study

In the period between April 2020 and December 2022, 638 children, belonging to economically and socially disadvantaged families, were welcomed at the healthcare center of the Solidarity Medicine Institute, guaranteeing them first-aid health assistance, for reducing the number of emergency visits and the possible overload due to limited available resources. Any request for assistance was accepted, as no selection criteria (medical or socio-economic) were adopted. At the first clinic visit, informed consent was obtained from the parents or the adult in charge of the pediatric patient for the collection of personal, clinical, and anamnestic data. Moreover, information about the socio-economic status and habits of the family was also recorded. Such information was stored in an electronic database (Excel, Microsoft Office package), employing a dedicated computer with electronically controlled access. The clinical documentation of each patient was collected in a completely anonymous form and registered in the computerized medical records for healthcare purposes. As defined by the Steering Committee of the Solidarity Medicine Institute, informed consent was prepared in accordance with the ethical principles for medical research involving human subjects following the Declaration of Helsinki and in accordance with the Italian national guidelines for privacy protection in health care. The Steering Committee further established that informed consent should be obtained for each visit to the care services of the Solidarity Medicine Institute. For minors, informed consent was obtained from their parents or a person exercising parental authority. Data and information were recorded and sorted following the indication of privacy protection as defined by the Italian law 675/96 art.13. The retrospective analysis of the data was performed between October and November 2024, to describe the socio-anthropometric and clinical features of this unique cohort of pediatric patients.

### 2.3. Pediatric Health Assessment

Each child was evaluated for their health status, and a dietary supplement was offered to each family to supplement protein and vitamin intake.

Pediatric check-ups were performed by a medical team composed of a pediatrician, a nurse, and a cultural mediator/educator. The pediatric examination, which lasted 20–30 min, monitored the bio-parameters for the standard assessment of health status such as weight, height, vital parameters (breath, heartbeat, etc.), blood pressure, and nasal and ear inspection. For infants (<=12 months old), weight was measured with an infant scale, while height was measured with a measuring tape. Moreover, their skull circumference was measured using a craniometer for children up to 20 months of age. Weight and height for children older than 1 year were measured on a standard scale combined with a stadiometer. The analysis of weight and height percentiles was performed for children < 2 years old, while Body Mass Index (BMI) was calculated for children aged 2 to 18 years.

The Junior-Bit 7 software (So.Se.Pe s.r.l., Padua, Italy) was employed to register clinical data and calculate percentiles and BMIs based on the World Health Organization information [18,19].

### 2.4. Psychiatric Evaluation

A team composed of a neuropsychiatrist, clinical psychologist, and cultural mediator interviewed the children who presented any signs of discomfort or distress and/or were previously diagnosed with a psychological condition by the public health service. Psychiatric evaluation was defined according to DSM-5 criteria [20]. The psychiatrist administered a series of graphic tests (writing, drawing, and construction tasks) and conducted interviews with both the child and the parents (always in the presence of an assisting psychologist). The assessments were conducted by a child neuropsychiatrist and a clinical psychologist specializing in child education and development. This dual independent evaluation ensured a solid framework for discussing emerging critical issues and verifying concordances across assessments. The administered tests were as follows: Draw-a-Person Test (DAP) [21], Pencil Test, Graphic Abilities Assessment Test (GAT), and Children’s Apperception Test (CAT) [22]. The developmental psychologist particularly focused on the administration of tests as such as the Wechsler Intelligence Scale for Children (WISC) [23], Child Behavior Checklist (CBCL) [24], Child Depression Inventory (CDI) [25], Bayley Scales of Infant and Toddler Development [26], and Revised Children’s Anxiety Scale (RCMAS) [27].

No strictly standardized tests were followed due to the COVID-19 restrictions, difficulties in language comprehension, and low literacy levels. However, both standardized and non-standardized tests were administered with a degree of flexibility, while maintaining a consistent setting for all participants. This setting included the same room, the same administration duration, the absence of distracting stimuli, and a fixed execution time. Regarding the DSM-5 framework, evaluations were initially conducted by psychologists and neuropsychiatrists from the Local Health Authority (ASL), thus integrating regional healthcare services. Subsequently, additional assessments were performed by the child neuropsychiatrist, yielding an inter-rater reliability index (Cohen’s Kappa Coefficient) of 0.70, indicating a “good” agreement level according to the Landis and Koch scale [28].

## 3. Results

### 3.1. The Solidarity Medicine Institute’s Primary Care Center and Pediatric Patient Population

During the COVID-19 pandemic period, between April 2020 and December 2022, we welcomed 638 pediatric patients at our primary healthcare center of the Solidarity Medicine Institute. There were 286 females (44.8%) and 352 males (55.2%) aged between 0 and 18 years. Some of them did not go to school. In terms of nationality, almost all the children (593, 93%) belonged to foreign families coming from 34 different countries, while the remaining 7% (45 patients) were Italians.

**Figure 2:**
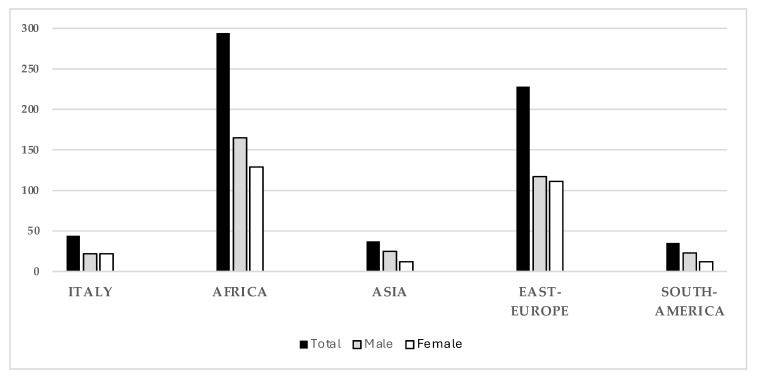
The socio-demographic data of the pediatric patients that accessed the street clinic units for their healthcare needs. Histograms represent subject distribution on the basis of the country of origin, indicating the total number (black) and the number of male (gray) and female (white) patients.

A vast majority (46%) of these children were from African countries (294), and 35.8% were from Eastern European countries (228). Furthermore, three other groups based on nationality were identified, i.e., those from Asian countries (37; 5.8%), those from South American countries (35; 5.5%), and Italians (45; 7%) (Figure 2).

In the Italian group, the male and female populations were equal (22 each), whereas in all the other groups, the number of male pediatric patients was higher than female pediatric patients.

The distribution of each country of origin for both males and females is reported in Supplementary Table S1. Nigeria was the country with the highest patient number among all the countries, while the highest patient number from a European country was from Romania.

By analyzing the age of the pediatric patients at the first visit (Supplementary Table S2), it was observed that the patients aged <2 years represented 12% of the entire patient population, while 50.81% (321/638) of patients were aged between 0 and 5 years. This is a particularly delicate age, when access to care is of primary importance for assessing the pediatric patient’s general condition, treating and preventing any pathology, and monitoring the patient’s growth. Moreover, free health care should be guaranteed by primary care pediatricians to each child in the first year of life; however, these children did not have a referred pediatrician. A total of 186 patients (29.1%) were aged between 6 and 10 years, corresponding to the age of elementary-school-going children, while 40 patients were aged between 11 and 16 years, corresponding to the age of children attending the last years of compulsory schooling. Only 2.6% of the pediatric cohort (18 pts) were over this limit (Supplementary Figure S1). All children of school-going age were unable to take advantage of school meals during the lockdown, a factor that further contributed to the risk of malnutrition in the enrolled population.

### 3.2. Socio-Cultural Characteristics of the Family Environment

When the children came for a visit, a questionnaire about the socio-economic background of the family was administered, and several types of information such as origin, religious habit, and working status were collected.

**Figure 3:**
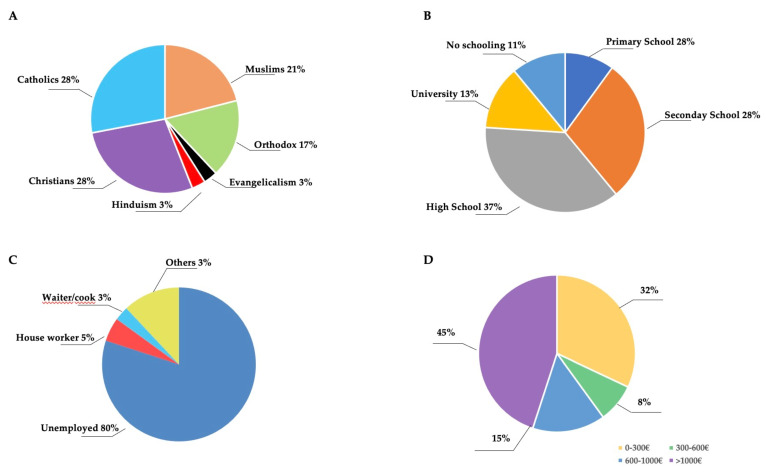
The socio-economic and cultural backgrounds of the families of pediatric patients enrolled in this study. The pie charts show the proportion of the following: (**A**) family religious tradition; (**B**) education level of the mothers; (**C**) working condition of the mothers; and (**D**) overall income of the family.

Regarding the family’s religious affiliation, although the percentage of Protestant Christians was the highest (31%), the other Christian denominations were also well represented (28% Catholic and 17% Orthodox), along with 21% Muslims and 3% Hindus, which indicates that the families felt welcomed and not discriminated against due to their ethnic and/or religious differences (Figure 3A).

Most of the parents were unemployed at the time of the first visit. The parental interviews were mostly administered to the mothers who had taken charge of the family care during the pandemic. For a subgroup of mothers (209), it was possible to define the cultural levels. Despite 50% of parents having a high school or university education level of education, 80% of the interviewed parents were unemployed (Figure 3B,C). Indeed, the socio-economic scale (ISEE) assigned to each family indicated that 55% of the families had an ISEE EUR <1000 (Figure 3D). The loss of work due to the pandemic also entailed a loss of registration for those who were entitled to it, breaching article 32 of the Italian Constitution, which guarantees the right to free treatment to indigent people.

### 3.3. Pediatric Healthcare Demands and Management

**Figure 4:**
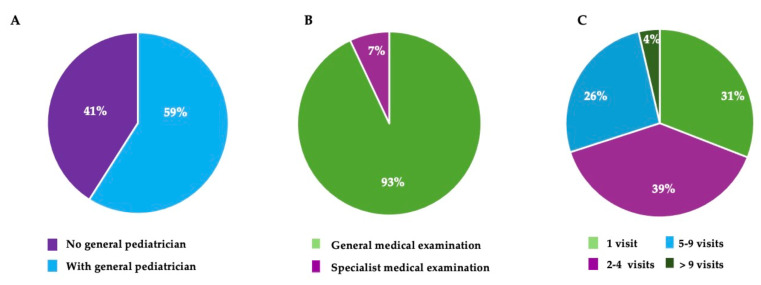
Pediatric patient information and distribution. The pie charts represent the proportion of the following: (**A**) pediatric patients having (sky blue) or not having (deep purple) a general pediatrician from the public National Health Services; (**B**) the reasons for a visit to the clinic street unit: general or specialist medical examination (green and deep purple, respectively); and (**C**) the distribution of the pediatric population based on the number of visits carried out.

A total of 638 pediatric patients had access to our clinic unit during the 2 years of the pandemic. Although most of the families had lived in Italy for over 5 years, 41% of their children were not registered with the national health system (Figure 4A).

In fact, registration is only possible for foreigners who have a regular presence and work in Italy. On the other hand, 59% of the patients, despite officially having a pediatric practitioner, came to our street clinic unit. This was due to the difficulties in contacting their pediatricians during the lockdown. In addition, food supplements, drugs, and hygiene kits that were necessary for the containment of the infection were freely distributed at the center.

Several visits were made by each patient, totaling 2300 visits. The main reason for the visit was to obtain a free consultation from a medical doctor on the basis of specific symptoms or for a check-up (Figure 4B). These visits were predominantly for children aged >5 years. The most common pathologies were flu (11%), gastrointestinal and respiratory pathologies (14 and 44.7%, respectively), and vitamin deficiency (9%). Most of the patients had one to four visits during this length of time (Figure 4C). Free visits and the distribution of free drugs facilitated the return of the pediatric patients for subsequent follow-ups and for continuing their treatment.

### 3.4. Clinical Profiles of the Pediatric Population

**Figure 5:**
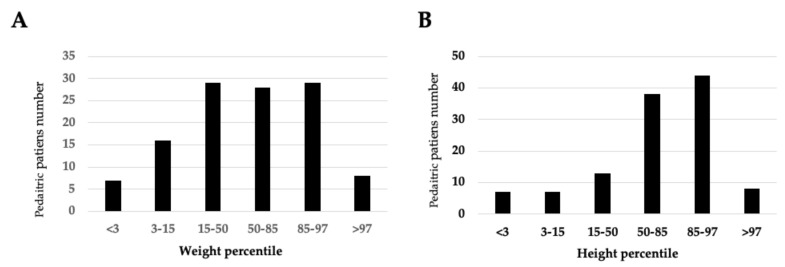
(**A**) Weight parameters of the pediatric cohort aged < 2 years; (**B**) Height parameters of the pediatric cohort aged < 2 years.

Weight and height were collected for each child, and BMI was assessed for children older than 2 years. The average weight of children aged 0–1 year (117 infants) was 9.36 kg (range: 2.81–18 kg), and their average height was 73.14 cm (range: 49–94 cm). Seven infants (5.7%) were below the 3rd weight percentile, while 28.9% (35) off infants exceeded the 85th weight percentile (Figure 5).

For the pediatric population aged 2 years and older, we also evaluated BMI as a bio-parameter. Table 1 shows the average weight and average height by age of 521 pediatric patients aged 2–18 years.

**Figure 6:**
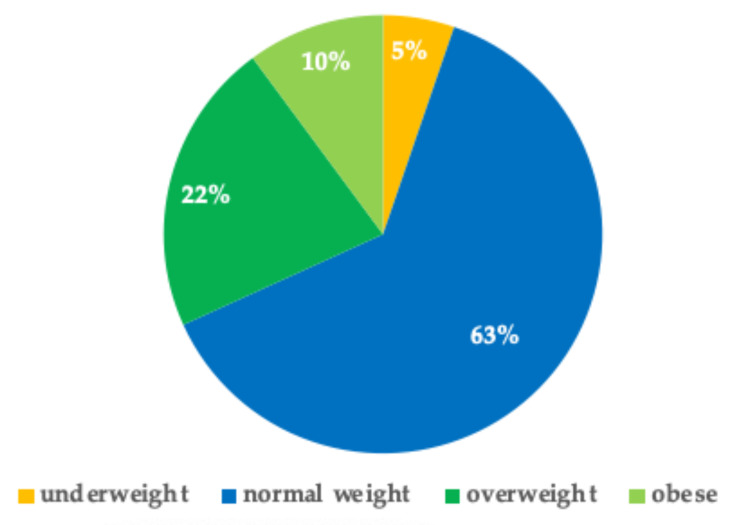
Distribution of pediatric patients in relation to BMI.

By analyzing the BMI of this group of patients, it was observed that a majority of children were in the highest percentiles of BMI. A total of 136 children were overweight and 63 were obese (21.7% and 9.5%, respectively) (Figure 6).

This is probably due to the higher proportion of children of African and Eastern European origin in the study group, as they tend to be in the upper percentiles.

Weight gain in children has been associated with the COVID-19 pandemic, due to the negative effects of restrictions: the lack of physical activity, the excessive intake of food, and the lack of stimulation.

Children from low socio-economic backgrounds were particularly affected, as they were often already overweight due to an unbalanced diet and an excessive intake of junk food. Few children (26.4%), in particular infants aged 2–5 years, were underweight.

### 3.5. Psychiatric Perturbation in Pediatric Patients

During the period that we considered, we confirmed 69 cases of psychiatric disorders among the children who accessed the healthcare system of our street clinic. The children were previously diagnosed by the Local Health Authority (ASL); however, during the pandemic, they did not have access to the required and necessary support, and they were therefore evaluated by the Solidarity Medicine Institute team. Among the 69 patients with psychiatric disorders, 41 patients were male (59.4%) and 28 were female (40.6%). The most represented nationality groups among these children were South American, corresponding to 43.5% (30 pts), and Italian, corresponding to 15.9% (11 pts). Moreover, 13% of the patients were from African countries, 11.6% were from Asian countries (eight patients), and 10% were from Eastern European countries (seven patients). Two patients were originally from EU countries, while another two patients were stateless (Supplementary Table S3).

The high prevalence of Latin American patients in this study was due to the high proportion of this community residing in this area of Rome. Forty pediatric patients (56%) were born between 2013 and 2017 (Table 2). A majority of the first psychiatric evaluations at the Solidarity Medicine Institute were performed in 2021 and 2022 (52% and 34.8% of visits, respectively), corresponding to the pandemic peaks (Table 2).

Interesting observations were made when this patient subset was evaluated based on the age of first diagnosis.

Twenty-two of the diagnosed children were aged between 2 and 5 years (32%), while thirty-nine of these children were aged between 6 and 10 years (56.5%). Furthermore, the street clinic service could individually support eight diagnosed children between 11 and 15 years of age (11.5%) (Supplementary Table S4). Table 3 shows the diagnosed psychiatric disorders: the most frequent disorders were Language Disorder (LD), diagnosed in 22 cases (30.6%), Specific Learning Disorder (SLD) (11 cases, 20.8%), and intellectual disability (10 cases, 13.9%). The other diagnoses were Attention Deficit Hyperactivity Disorder (ADHD) (8), autism (3), enuresis (1), selective mutism (1), Oppositional Defiant Disorder (ODD) (4), Generalized Anxiety Disorder (GAD; 1), Sleepwalking (1), movement disorder (4), and depression (2) (Table 3). No evidence of prenatal alcohol exposure was reported in any of the identified cases.

This indicated that the school as well as pediatric monitoring had been inefficacious at intercepting the signs of discomfort and disease due to the suspension of educational and health assistance services during as well as before the pandemic.

## 4. Discussion

During the pandemic, there was an almost complete shutdown of vital child health and welfare services, including nutritional programs, maternal and newborn care, immunization services, and community-based child-protection programs. It is evident that racial/ethnic minority groups and those with a low socio-economic status are more vulnerable to COVID-19; therefore, public health policymakers, practitioners, and clinicians should be aware of these inequalities and strive to narrow the gap by focusing on vulnerable populations [29]. The COVID-19 pandemic is expected to increase the risk of all forms of malnutrition, in particular for socially and economically disadvantaged people. Several studies have shown that a low income during the pandemic was correlated with a greater risk of compromised maternal and child health [30,31,32,33,34].

In this retrospective observational study, we describe the healthcare needs of a fragile pediatric population in a disadvantaged suburb of Rome metropolitan city during the pandemic and the response provided by the primary healthcare center of the Solidarity Medicine Institute, substituting for the completely lacking public health and social services.

Children who live and grow up in disadvantaged neighborhoods are likely to have inadequate health care, a higher risk of contracting acute and/or chronic diseases, and a lower quality of life [29,35].

During the pandemic, the fragmentation of the Italian system meant a lack of support for vulnerable groups as the pandemic gained ground [36]. The lockdown had worsened social isolation and health disparity. The healthcare system was unable to cater to patient requests during the pandemic, and the emergency also made it difficult for community services to provide social support to the most vulnerable sections of the population [37]. Indeed, the Solidarity Medicine Institute provided primary health care to children who had been officially registered with a general pediatrician from the National Health Service (59%), but they could not receive any service during the pandemic. The outreach clinic was able to intercept health and social needs independent of the religious, ethnic, and educational background of the families.

Furthermore, the loss of a job and a low/absent income are two key factors that should be considered. It is well established that socio-economic poverty profoundly affects children’s subjective well-being and mental health. In fact, inequalities of social and economic origin do not only emerge as risk factors but also as actual etiological/causal determinants of health [38,39]. This is true for this study’s population since 80% of the surveyed population was jobless, 55% of families had an ISEE (economic parameter to define economic wellness) of less than EUR 1000, and 11% of children (69/638) had psychiatric disorders. Access to employment opportunities is also low in the suburbs of Rome with only 3.7% of college graduates and 21% of high-school graduates [40], and our study population resided in a municipality with the lowest average income in the entire Rome metropolitan city [41].

In this context, the real-world experience of the Solidarity Medicine Institute resembles that of safety-net healthcare systems in the USA. They are health centers that provide essential health services to uninsured/underinsured people. The COVID-19 pandemic has shown that these systems have been critically important in the management of the pandemic and the implementation of health care during the pandemic for vulnerable communities [42]. Noteworthily, the age of 50% of the pediatric population was between 1 and 60 months, an age range that is crucial for health development and future wellness. This retrospective observational study also considered the weight/height balance: we found that 22% of the children were overweight, and 10% were obese, while 5% were underweight. The data of a cohort of 46,173 children (8–9 years) from Italy collected in 2019 showed that at the national level, 29.8% of children were overweight and 9.4% were obese [43], while for the central regions (where Rome is located), the incidence of obesity was 8%. Indeed, it has been documented that the incidence of being overweight and obese varies depending on the Italian region, i.e., there is a lower incidence in Northern Italy and an increased incidence in Southern Italy [43,44]. Data from our pediatric cohort appear to be in line with the national figures, but the obesity incidence appears to be higher than the regional cut-off value (10% vs. 8%).

However, several factors hamper this analysis by not providing conclusive findings: the size of the cohort, the different age ranges, the absence of information about ethnicity, and the period of the study. There is a consensus that the COVID-19 pandemic dramatically modified children’s nutrition habits, increasing concerns about both undernutrition and obesity [45,46]. These changes were related to other factors beyond food intake. Housing restrictions, physical inactivity, and the absence of positive stimuli and social interaction due to the absence of the school environment had dramatic effects on children, and the effects were possibly amplified in vulnerable children [47,48]. This was particularly critical for those children that already had behavioral and psychiatric disorders.

In this regard, the Solidarity Medicine Institute was effective in supporting such pediatric patients. Indeed, 69 children arrived at the outreach clinic with psychiatric disorders already monitored by the Local Health Authority (ASL), who were left alone with their families without any medical and psychological support for the children and for the parents/caregivers. The psychiatric team took charge of the care of these children, again performing psychiatric evaluation to define the optimal intervention strategy feasible in that period, despite the COVID-19 restrictions. The children with psychiatric and behavioral disorders represented 10.8% of the entire population of this retrospective observational study. According to the Italian national institute of health (ISS), in 2022, childhood and adolescent neuropsychiatric disorders in Italy were on the rise, affecting nearly 2 million children and adolescents aged 0–17 years, representing 10–20% of this age group [49], and clearly COVID-19 has been a triggering factor [50]. However specific information about the incidence of such disorders in vulnerable children and adolescents is lacking. The results of this study may offer a relevant snapshot of neuropsychiatric disorder incidence in vulnerable and immigrant communities, which is otherwise difficult to obtain and may remain underestimated.

One significant observation was that there was a notably high number of children diagnosed with Specific Language Disorder (SLD) and Specific Learning Disorder (SLD). Interestingly, there was a low incidence of Oppositional Defiant Disorder (ODD). Cultural background and integration within the local community may be two relevant variables to consider when analyzing the data. Indeed, families experience difficulty in providing tools to enable their children to integrate and be included in the community that they reside in. In terms of linguistic expression difficulties, children may develop early and debilitating forms of learning disorders [51,52] that, in addition to being linked to biological factors [53], may also be associated with intra-family challenges pertaining to the assimilation and provision (by parents or guardians) of the basic skills necessary for interacting with others. This suggests that, in this population sample, one triggering factor for such disorders could be the inability of family members to assist their offspring in social inclusion or, conversely, their choice to deny them the opportunity to integrate into a new culture in addition to their own. Indeed, relationships are crucial for children’s development as well as for adults. In fact, well-being is related to a multiplicity of qualitative factors (educational, cultural, and relational) that, while they are not immediately visible and are more difficult to measure, may produce destructive effects when they are not considered or are even denied. This is the concept of vital poverty, i.e., the “Poverty of relationships, affective poverty, emptiness of meaning, fall of values, loss of moral and religious sense are the indices of this new form of poverty […] and constitute a risk factor, a substrate of psychopathological vulnerability” [54].

In this regard, the Solidarity Medicine Institute, as well as other primary healthcare and socio-educational services, act not only as the providers of health assessments, therapeutic interventions, and food support, but also aid in mending the laceration of social relationships and health care. The street clinics compensated for the failure of the healthcare system, offering a proactive service to its users. They offered relational and organizational contexts for people who could not obtain public health support as well as for those who could not access health care despite being officially registered with a pediatrician, and these patients preferred to come to the street clinics due to the ease of contact and the provision of drugs and life-related support. These activities mitigated health and social inequalities [55,56], which are risk factors for violence against children recognized by the WHO [57]. Furthermore, we considered the determinants of physical and psychological health in childhood and adolescence. We detected mental and physical distress in children from disadvantaged families during the pandemic. For this reason, we inferred that the pandemic showed us a reality that would have otherwise remained hidden. All of the families that received social and nutritional support, along with psychiatric diagnoses, for their children during the pandemic now follow a rehabilitation program at the Solidarity Medicine Institute.

## 5. Conclusions

This retrospective observational study described the healthcare needs of a fragile pediatric population in a disadvantaged suburb of Rome during the COVID-19 pandemic and the central role played by the healthcare primary center of the Solidarity Medicine Institute in promptly responding to these needs. Indeed, the presence of primary healthcare services that are free and easy to access has been pivotal in limiting the health and social disparity gap created by the pandemic in a disadvantaged urban area. This study underlines the strategic function of outreach clinics in safeguarding health care and preventing disease in disadvantaged patients as well as in the whole community by constructing and amending the community social network, especially during a global health crisis.

We believe that this study is extremely useful in defining novel and efficacious strategies of interventions in a large Western city to counteract healthcare and “vital poverty” needs and guarantee the fulfillment of Sustainable Development Goal numbers 3 and 10, i.e., *no one is left behind* [13].

## Figures and Tables

**Table 1:** Distribution of pediatric patients aged 2–18 years based on their weight, height, and BMI.

	Females				Males			
Age (Years)	Weight *	Height *	BMI *	BMI Range	Weight *	Height *	BMI *	BMI Range
**2**	14.1	92.7	16.52	13–19.9	14.1	91.7	16.8	13.7–19.9
**3**	15.7	99.7	15.94	11.5–18.7	17	103.8	15.8	11–19.6
**4**	18.6	109.9	15.3	10.2–18.4	19.5	111.1	15.8	12.5–18.6
**5**	22.5	118.3	16	13.1–20.4	22.4	117.4	16.3	12.2–20.4
**6**	27	123.3	16.48	12.2–20.3	24	122.6	16	12.3–21.6
**7**	26.1	125.4	16.6	13–20.7	29.1	127.2	17.8	12.6.22.3
**8**	29.6	131.9	16.9	9.8–20.45	30.8	129.5	18.3	14.7–21.2
**9**	34.5	134	19.3	16.2–24.3	33	135.7	17.6	15.3–21
**10**	38.8	138.4	20.14	16.5–24.7	39.3	145.2	20.2	15.8–21.2
**11**	40	144.4	18.37	16.4–20.6	43.7	147.6	20	16.8–26
**12**	50.7	156.57	20.2	16.5–26.7	55.1	154.5	22.5	16–35.9
**13**	55.6	160	21.5	16.2–27.9	54.4	161.1	20.9	15–24.2
**14**	59.4	160	23.3	19.6–27.5	53.4	164.2	19.9	17.3–22
**15**	48.7	157.4	19.7	19–21.2	57.4	168.2	20.1	29–22.7
**16**	55.6	165.6	20.2	17.6–24.6	59	170.8	21.4	18.3–23.3
**17**	-	-	-	-	66.6	177	23.2	20–27.6

* indicates the average value.

**Table 2:** Year of birth and year of diagnosis.

Year of Birth	Total	M	F
2006	1	0	1
2008	2	2	0
2009	2	0	2
2010	2	2	0
2011	3	2	1
2012	5	5	0
2013	10	4	6
2014	9	5	4
2015	9	6	3
2016	4	1	3
2017	8	7	1
2018	4	4	0
2019	4	2	2
2020	3	1	2
2021	2	0	2
Year of diagnosis	Total	M	F
2021	36	22	14
2022	24	14	10
2023	9	6	3
Total	69	42	27

**Table 3:** Psychiatric disorders diagnosed in the pediatric population during the COVID-19 pandemic.

**Disorder**	**Number of Cases**	**%**
LD	22	30.6
SLD	15	20.8
ID	10	13.9
ADHD	8	11.1
ODD	4	5.6
Movement disorders	4	5.6
Autism	3	4.2
Depression	2	2.8
Enuresis	1	1.4
Selective mutism	1	1.4
GAD	1	1.4
Sleepwalking	1	1.4

## Data Availability

The original contributions presented in this study are included in the article/Supplementary Material. Further inquiries can be directed to the corresponding author(s).

## Supplementary Materials

The following supporting information can be downloaded at: https://www.mdpi.com/article/10.3390/children12040443/s1, Supplementary Figure S1: Distribution of the patient population on the basis of school frequency. Histograms represent subject distribution on the basis of age range: the total (black), female (grey) and male (white) numbers of patients; Supplementary Table S1: Country of origin and number of patients; Supplementary Table S2: Age distribution of pediatric patients at the 1st visit; Supplementary Table S3: Family country of origin for psychiatric patients; Supplementary Table S4: Age at the psychiatric diagnosis.

## Abbreviations

The following abbreviations are used in this manuscript:

BMI	Body Mass Index
DSM-5	Diagnostic and Statistical Manual of Mental Disorders 5
DAP	Draw-a-Person Test
GAT	Graphic Abilities Assessment Test
CAT	Children’s Apperception Test
WISC	Wechsler Intelligence Scale for Children
CBCL	Child Behavior Checklist
CDI	Child Depression Inventory
RCMAS	Revised Children Anxiety Scale
LD	Language Disorder
SLD	Specific Learning Disorder
GAD	Generalized Anxiety Disorder
