# Supplementary material for: Assuring Primary Healthcare Services to Vulnerable Children in a Disadvantaged Suburb of Rome Metropolitan City During the Pandemic: Responses to the Crisis
Source: Children (Basel). 2025 Mar 30;12(4):443. doi: 10.3390/children12040443 (PMC12025899; doi:10.3390/children12040443)
Supplement: Supplementary file 1 [file children-12-00443-s001.zip › children-3504388-supplementary.pdf]

Assuring primary healthcare services to vulnerable children in a disadvantaged suburb of Rome metropolitan City during pandemic: lessons from the crisis

Rughetti Aurelia, Buonomini Anna Rita, Russo Leonardo, Mazzoli Francesca, Urbano Suleika, Iordanoglou Fotinì, Palagiano Cataldo, Barletta Manuel, Casartelli Samuele, Aldo Morrone, Ercoli Lucia

Supplementary Tables and Figures

Supplementary Table S1 - Country of origin and number of patients

Table S.1 - Country of origin and number of patients				
Geographic Area		Total	M	F
	Italy	44	22	22
Africa				
	Algeria	4	1	3
	Angola	3	1	2
	Ivory Coast	3	1	2
	Egypt	14	11	3
	Eritrea	1	1	0
	Ethiopia	5	1	4
	Ghana	4	3	1
	Guinea	2	1	1
	Kenya	1	1	0
	Morocco	53	28	25
	Nigeria	176	102	74
	Senegal	12	4	8
	Togo	4	1	3
	Tunisia	9	7	2
Asia				
	Afghanistan	2	2	0
	Bangadlesh	13	7	6
	India	18	14	4
	Israel	1	0	1
	Mali	1	1	0
	Pakistan	3	0	3
	Sri Lanka	1	1	0
East-Europe				
	Albania	31	14	17
	Bosnia	22	14	8
	Bulgaria	1	0	1
	Montenegro	32	20	12
	Romania	104	47	57
	Serbia	28	14	14
	Ukraine	11	9	2
South-America				
	Colombia	4	3	1
	Ecuador	2	2	0
	Honduras	3	2	1
	Peru'	26	17	9

Supplementary Table S2 - Age distribution of pediatric patients at the 1st visit

Table S.2 - Age distribution of the pediatric patients at the 1st visist				
**YEARS AT 1st VISIT**	**TOTAL**		**MALE**	**FEMALE**
	**Nr patients**	**% total population**	Nr	Nr
**0-1**	75	11,75	41	34
**1**	46	7,21	22	34
**2**	63	9,9	36	27
**3**	51	8	26	25
**4**	44	6,9	22	22
**5**	45	7,05	22	23
**6**	40	6,3	22	18
**7**	50	7,83	25	25
**8**	38	5,95	21	17
**9**	37	5,8	17	20
**10**	21	3,3	12	9
**11**	25	3,9	18	7
**12**	27	4,23	20	7
**13**	29	4,6	17	12
**14**	12	1,9	5	7
**15**	9	1,41	5	4
**16**	8	1,25	5	3
**17**	7	1,1	6	1
**18**	11	1,5	10	1
**TOTAL**	638	99,88	352	296

SupplementaryTable S3 - Family country origin of psychiatric patients

Table S.3 family country origin of psychiatric patients			
Family country origin	**Total**	**M**	**F**
Italy	11	6	5
**Europe**	**2**	**1**	**1**
Albania	1	0	1
Spagna	1	1	0
**Est-Europe**	**7**	**4**	**3**
Romania	6	4	2
Serbia	1	0	1
**Africa**	**9**	**4**	**5**
Algeria	1	0	1
Egitto	1	0	1
Marocco	4	2	2
Nigeria	2	1	1
Tunisia	1	1	0
**Asia**	**8**	**5**	**3**
Turchia	1	1	0
Bangladesh	6	4	2
Pakistan	1	0	1
**South-America**	**30**	**19**	**11**
Bolivia	4	2	2
Colombia	1	1	0
Equador	2	1	1
Perù	21	13	8
Uruguay	2	2	0
**Stateless**	**2**	**2**	**0**
Stateless	2	2	0

Supplementary Table S4

Table S.4. Age at psychiatric diagnosis		
**Age (years)**	**M**	**F**
**2**	1	4
**3**	6	1
**4**	5	0
**5**	2	3
**6**	4	2
**7**	8	4
**8**	4	6
**9**	5	2
**10**	3	1
**11**	3	0
**12**	0	2
**13**	0	2
**15**	1	1

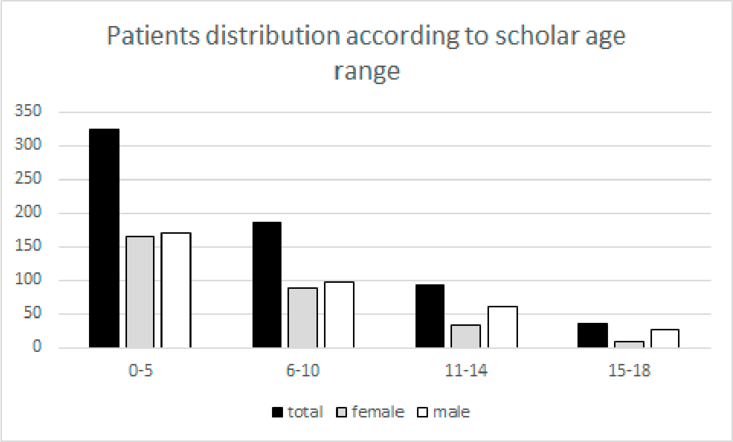

Supplementary Figure S1– Distribution of the patient population on the basis of school frequency. Histograms represents subject distribution on the basis of age range: the total (black), female (grey) and male (white) number of patients.
